# Antiplatelet Monotherapies for Long-Term Secondary Prevention Following Percutaneous Coronary Intervention

**DOI:** 10.3390/jcm14155536

**Published:** 2025-08-06

**Authors:** Claudio Laudani, Daniele Giacoppo, Antonio Greco, Luis Ortega-Paz, Georges El Khoury, Davide Capodanno, Dominick J. Angiolillo

**Affiliations:** 1 Division of Cardiology, Azienda Ospedaliero-Universitaria Policlinico “Rodolico—San Marco”, University of Catania, 95124 Catania, Italy; claudani313@gmail.com (C.L.); giacoppomd@gmail.com (D.G.); a.greco90@gmail.com (A.G.); dcapodanno@gmail.com (D.C.); 2 Division of Cardiology, University of Florida College of Medicine, Jacksonville, FL 32209, USA; luis.ortega@jax.ufl.edu (L.O.-P.); georges.elkhoury@jax.ufl.edu (G.E.K.)

**Keywords:** coronary artery disease, ischemic heart disease, monotherapy, DAPT, bleeding

## Abstract

In patients with coronary artery disease (CAD) undergoing percutaneous coronary intervention (PCI), antiplatelet therapy is the cornerstone of treatment for secondary prevention. Although dual antiplatelet therapy (DAPT) consisting of aspirin and a P2Y_12_ inhibitor is the current standard of care, being, respectively, recommended for 6 and 12 months in patients with chronic and acute coronary syndrome without a need for oral anticoagulation, the continuous improvement in PCI technology and pharmacology have significantly reduced the need for long-term DAPT. Mounting evidence supports the administration of P2Y_12_ inhibitor monotherapy, particularly ticagrelor, after a short period of DAPT following PCI as a strategy to reduce bleeding without a trade-off in ischemic events compared to standard DAPT. In addition, there is a growing literature supporting P2Y_12_ inhibitor monotherapy also for long-term secondary prevention of ischemic events. However, the data to this extent are not as robust as compared to the first-year post-PCI period, with aspirin monotherapy still remaining the mainstay of treatment for most patients. This review aims to summarize the rationale for long-term antiplatelet therapy, the pharmacology of current antiplatelet drugs tested for long-term administration as monotherapy, and current evidence on the available comparisons between different long-term antiplatelet monotherapies in patients with CAD.

## 1. Introduction

Coronary artery disease (CAD) is a complex entity characterized by atherosclerotic plaques, which progressively develop in the epicardial arteries, leading to myocardial ischemia [1]. CAD encompasses different clinical presentations, including chronic coronary syndrome (CCS) and acute coronary syndromes (ACSs) [1]. In this context, percutaneous coronary intervention (PCI) to treat coronary artery stenosis is recommended to improve outcomes in patients with ACS and to reduce symptom burden in CCS patients. However, given the complex and systemic nature of the disease, PCI by itself is not resolutive, and medical therapy should be titrated to improve short- and long-term outcomes [2,3].

Antiplatelet drugs play a pivotal role in the management of patients with CAD, particularly in those undergoing PCI, as the interplay between platelets and the arterial wall has been shown to be a pivotal driver of atherosclerotic plaque progression and periprocedural complications [4,5]. However, since the ischemic and bleeding risks vary over time after PCI, the intensity of the antiplatelet regimen should vary accordingly [6]. Indeed, a more intense regimen with contemporary administration of aspirin and a P2Y_12_ receptor inhibitor, known as dual antiplatelet therapy (DAPT), is warranted during the first 1–3 months after PCI, while administration of antiplatelet monotherapy is recommended thereafter to counterbalance the increased risk of bleeding induced from reduced platelet reactivity while maintaining substantial ischemic protection [2,3,7]. However, the optimal duration of DAPT and which antiplatelet agent should be administered as long-term monotherapy afterwards has been a topic of debate over the last two decades [8], with the role of aspirin as the backbone of secondary prevention being questioned by recent clinical trials investigating the role of P2Y_12_ receptor inhibitor monotherapy [9].

This review aims to summarize the rationale for long-term antiplatelet therapy, the pharmacology of current antiplatelet drugs tested for long-term monotherapy, and current evidence on the available comparisons between different antiplatelet monotherapies in patients with CAD.

## 2. Rationale for Long-Term Antiplatelet Therapy for Secondary Prevention After PCI

Arterial thrombosis is a complex and dynamic process triggered by the contact between the bloodstream and the injured vessel wall or contact with a foreign body [10]. In this scenario, platelets have a primary role in arterial thrombosis, as the contact of the subintimal tissue with specific platelet receptors, as well as the amplification of thrombin activity on the platelet surface, are key in advancing thrombus formation [11,12].

**Figure 1:**
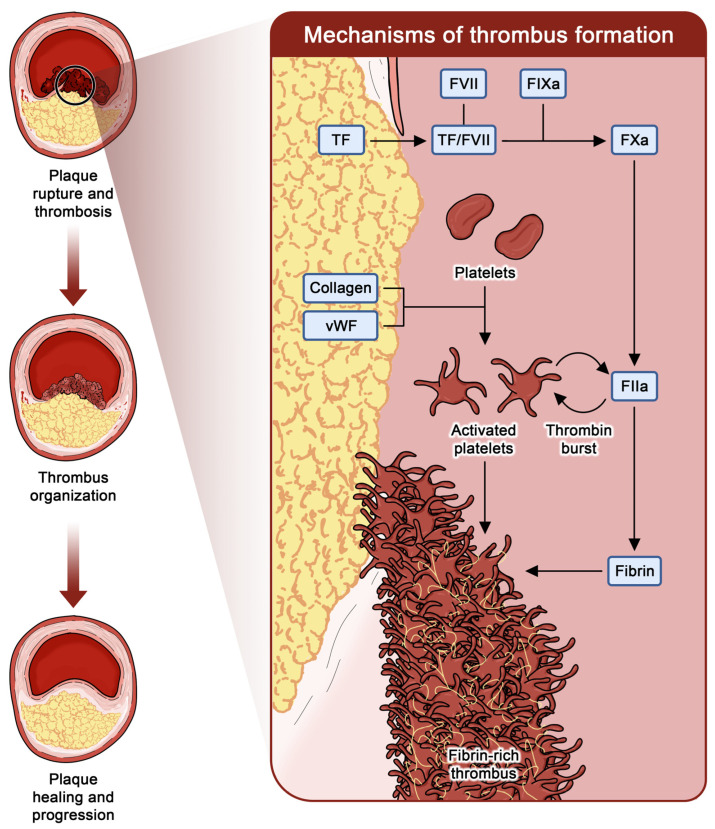
**Relationship between arterial thrombosis and plaque progression.** This figure illustrates the mechanisms of plaque progression over time (left) and thrombosis (right). Whenever the subendothelial material is exposed to the bloodstream, the interaction between collagen and vWF leads to platelet adhesion and activation. Platelet activation leads to changes in platelet shape and the expression of several receptors on the platelet surface, which further increase interactions with both subendothelial material and other platelets, leading to platelet aggregation. In addition, exposure of TF allows interaction with circulating FVIIa, setting off a chain of enzymatic reactions culminating in factor X activation. FXa is then able to convert factor II (prothrombin) into FIIa (thrombin) and subsequent formation of fibrin, leading to a fibrin-rich thrombus. Of note, platelets are activated by FIIa, and the activated platelet surface is able to produce large quantities of FIIa, in a complex interaction usually known as “thrombin burst”. The formed thrombus triggers a migration of inflammatory cells and smooth muscle cells, being responsible for the fibrin degradation and collagen production typical of thrombus organization, finally resulting in an additional layer of collagen that narrows the lumen. Abbreviations: FIIa, thrombin; FVIIa, activated factor VII; FXa, activated factor X; TF, tissue factor; vWF, von Willebrand factor.

Although often considered an acute event, arterial thrombosis often occurs without clear clinical manifestations, being part of the continuous evolution of atherosclerotic plaque development (Figure 1) [13,14]. Subclinical arterial thrombi trigger the migration of inflammatory cells and smooth muscle cells, being responsible for fibrin degradation and collagen production, ultimately resulting in an additional layer of collagen that narrows the lumen, being the landmark of plaque progression [15]. Therefore, antiplatelet therapy has a central role across the whole spectrum of CAD management, being able to reduce periprocedural and short-term PCI complications as well as modulating long-term progression of the disease [5,16].

Antiplatelet drugs have the major drawback of bleeding, which can be as harmful as a repeated myocardial infarction on overall patient prognosis [17], even when happening during the index hospitalization [18]. The availability of stent platforms with thinner struts and overall less thrombogenic profiles, and the recognition that ischemic and bleeding risk have different evolutions over time, has led to the overall transition from more intense and prolonged DAPT regimens to shorter DAPT regimens followed by antiplatelet monotherapy [5,6,19,20]. In addition, while aspirin has been the cornerstone of secondary prevention in patients with CAD undergoing PCI, novel evidence has shown that P2Y_12_ inhibitor monotherapy may improve net clinical benefit compared to aspirin monotherapy in selected populations. To this extent, several antiplatelet treatment regimens have been evaluated for secondary prevention after PCI [5].

## 3. Pharmacology of Oral Antiplatelet Agents Recommended for Secondary Prevention in Patients with CAD

Platelet-activated thrombosis is characterized by three major steps, namely, the initiation, extension, and perpetuation phases [11]. During the initiation phase, platelets adhere to the injured vessel wall through interaction between platelet glycoprotein IIb/IIIa, circulating von Willebrand factor, and the vessel’s collagen matrix [21]. Once activated, platelet and endothelial cells release different platelet-activating factors, including thromboxane A_2_, adenosine diphosphate (ADP), and thrombin, leading to additional recruitment and the aggregation of nearby platelets during the extension phase [22]. At this point, the large platelet surface serves as a catalyzer for thrombin generation, resulting in a thrombin burst and significant production of fibrin during the perpetuation phase, ultimately leading to clot formation and stabilization [12,23]. Currently approved oral antiplatelets for the management of CAD mostly act during the extension phase, targeting the soluble agents responsible for platelet activation and aggregation. Such agents can be divided into two major pharmacodynamic classes, namely cyclooxygenase (COX) inhibitors and P2Y_12_ receptor inhibitors.

### 3.1. Cyclooxygenase Inhibitors

Acetylsalicylic acid (i.e., aspirin) is the oldest available antiplatelet agent and, accordingly, the one with the largest available evidence for its use. Aspirin acts by irreversible acetylation of COX enzymes, preventing the conversion of arachidonic acid to prostanoids and subsequent interaction with G-coupled receptors [24]. However, the distribution of COX enzymes varies among different tissues, with the COX-1 isoform being primarily expressed by platelets and involved in platelet activation, while COX-2 being mostly expressed by endothelial cells and other tissues and primarily involved in vascular tone regulation and nociception [24]. As aspirin shows a 100-fold higher affinity for COX-1 isoforms, doses of aspirin as low as 30 mg are sufficient to completely block platelet aggregation compared to the higher dosages needed to exert analgesic properties, for which 10-fold higher dosages are requested [25].

Although aspirin takes a relatively long time to reach peak concentration, its antiplatelet effect starts as soon as it reaches the portal circulation, inhibiting platelet reactivity even before being detectable in peripheral blood [24,25]. Similarly, although the drug exhibits a half-life of 20 min, the irreversible inhibition of the COX-1 enzyme and the inability of platelets to synthesize new proteins allow for the antiplatelet effect to last for the entire lifespan of the platelet (around 7 to 10 days) [25].

### 3.2. P2Y_12_ Receptor Inhibitors

ADP plays a central role in the initiation and extension phases of platelet aggregation and thrombus formation, mostly by interacting with P2Y receptors on the platelets’ surface. P2Y receptors are G-coupled receptors acting differently depending on the G-protein subtype associated, with P2Y_1_ and P2Y_12_ receptors being the main mediators of ADP effects on platelet activation [26]. The P2Y_1_ receptor is linked to Gq protein, being able to generate inositol triphosphate and stimulate intracellular calcium mobilization and having a central role in the initial platelet shape change and transient activation [27]. Conversely, the P2Y_12_ receptor has a more prominent role in platelet activation. Specifically, the P2Y_12_ receptor is coupled with a Gi protein, which, upon activation, inhibits adenyl cyclase, leading to reduced production of cyclic adenosine monophosphate and reduced activity of protein kinase A. Such a blockage results in the lower phosphorylation of vasodilator-stimulated phosphoprotein and subsequent platelet activation, stabilization, and paracrine release of thromboxane A_2_ and ADP [27,28,29]. Therefore, P2Y_12_ receptor inhibition has been associated with reduced activity of P2Y_12_ receptor signaling and other platelet activation pathways [29]. Two major classes of oral P2Y_12_ receptor inhibitors have been tested in the context of CAD, namely, thienopyridines and cyclopentyl-triazolo-pyrimidine [30].

Thienopyridines are pro-drugs that require metabolic conversion into the active metabolites [31]. The main activity of these agents is to irreversibly block the P2Y_12_ receptor through a disulfide bond between the thiol groups of the molecule and the receptor’s cysteine residue [32]. While the parent compound of this class, ticlopidine, has been largely discontinued due to significant hematologic toxicity, two other drugs of this class have been extensively tested, namely clopidogrel and prasugrel. Clopidogrel is a second-generation thienopyridine, which requires a two-step process involving hepatic cytochrome P450 (CYP) enzymes to be converted into the active metabolite [33]. After administration of a 300 mg loading dose, the antiplatelet effect is achieved within 90 min [34], leading to an average 18% reduction in platelet aggregation from baseline [35]. Like aspirin, the antiplatelet effect of the drug will last for the entire life span of the platelet, due to the irreversible binding of the active metabolites to P2Y_12_ receptors [36]. However, clopidogrel responsiveness is affected by significant interindividual variability, with as much as 30% of patients experiencing suboptimal antiplatelet effects, exposing them to an increased risk of thrombotic events [37,38]. The recognition of such a high degree of under-responsiveness prompted the investigation of alternative antiplatelet agents able to achieve more prompt, potent, and predictable antiplatelet effects.

Prasugrel is a third-generation thienopyridine developed to overcome the limitations of clopidogrel, being more potent and having lower interindividual variability. Indeed, prasugrel is converted by intestinal esterases into an intermediate form that will then be processed by hepatic CYP into the final active metabolite [31]. Compared with clopidogrel, the overall metabolism process is more efficient, allowing it to achieve significantly higher concentrations of the active metabolite as well as lower interindividual variability in pharmacodynamic responses [39]. After administration of a 60 mg loading dose, the antiplatelet effect is seen after 15 min and peaks within the first 2 to 4 h, resulting in a mean platelet inhibition of 41.6% from baseline, being 20% lower than that seen with clopidogrel [35]. Once again, the antiplatelet effect lasts for the entire lifespan of the platelet, given the irreversible blockage of the receptor.

Finally, the only cyclopentyl-triazolo-pyrimidine available is represented by ticagrelor, which has differences compared with thienopyridines. Specifically, ticagrelor does not require conversion into an active metabolite, being already active per se, and inhibits the P2Y_12_ receptor in a non-competitive fashion through allosteric modulation, interacting with a different binding site than ADP [39]. After oral administration of 180 mg, ticagrelor is rapidly absorbed, reaching maximum plasma concentration within 1.5 h [40]. Although the drug is already active, 30% of the drug is metabolized through hepatic CYPs into an active metabolite sharing similar antiplatelet effects to the parent compound [40]. The maximum antiplatelet effect is achieved within 2 h, being 30% more powerful than clopidogrel [41]. Both ticagrelor and the active metabolite show an estimated half-life of 12 h, requiring twice-daily administration to maintain steady-state concentration [42].

## 4. Antiplatelet Monotherapy Compared with Dual Antiplatelet Therapy After PCI

Although 12 months has been the standard DAPT duration for years, advancements in PCI procedures, including availability of less thrombogenic devices, better lesion selection and optimization [43,44,45,46], as well as increased awareness of the harmful effects of bleeding on overall prognosis, have questioned the need for such long-term DAPT, leading to the conduct of several trials evaluating shorter DAPT regimens in patients undergoing PCI (Table 1) [47].

**Figure 2:**
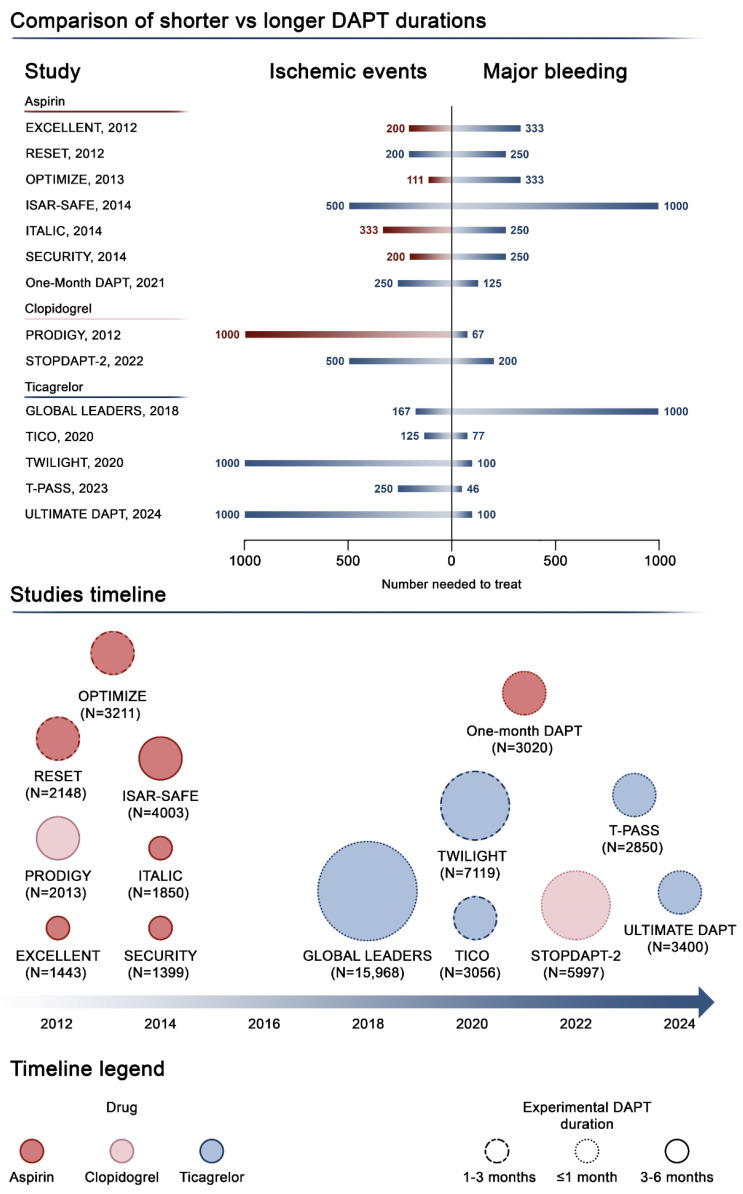
Antiplatelet monotherapy compared with dual antiplatelet therapy after PCI. This figure illustrates the current evidence on antiplatelet monotherapy compared with DAPT. The top panel shows the number needed to treat for benefit (blue bars) or harm (red bars) for ischemic endpoints and major bleeding across studies. The timeline at the bottom illustrates current available studies exploring shorter DAPT duration followed by antiplatelet monotherapy compared with longer DAPT duration. The dimensions of the circles are proportional to the number of patients enrolled, while colors highlight the specific antiplatelet agent administered as monotherapy, and the shape of the circumference represents the DAPT duration before monotherapy. Abbreviations: CI, confidence interval; DAPT, dual antiplatelet therapy; HR, hazard ratio [48,49,50,51,52,53,54,55,56,57,58,59,60,61].

Data from large-scale observational registries highlighted that shortening the DAPT duration is associated with improved outcomes across different subgroups, including both the general population [62,63] and patients with a high bleeding risk [64]. However, data from the PARIS registry also highlighted that abrupt DAPT discontinuation, namely, “DAPT disruption”, is associated with a higher risk of ischemic events, while careful baseline risk assessment and upfront planning of DAPT interruption by a physician should be the standard of care for optimizing DAPT titration [63,65]. As DAPT consists in the administration of aspirin and a P2Y_12_ receptor inhibitor, a key question has been which of the two classes should be withheld [66]. However, since aspirin is the drug with the largest amount of evidence supporting its use in CAD patients undergoing PCI, earlier trials evaluated the effects of aspirin monotherapy after a short course of DAPT (Figure 2).

### 4.1. Evidence and Recommendation on Short DAPT Followed by Aspirin Monotherapy

The first antiplatelet monotherapy strategy after a short course of DAPT to be compared with prolonged DAPT duration was represented by aspirin monotherapy.

The EXCELLENT trial was the first trial exploring the safety and efficacy of shortening DAPT duration, randomizing 1443 patients to 6- or 12-month DAPT, followed by aspirin monotherapy, and it found that the 6-month DAPT duration was non-inferior to 12-month DAPT for target vessel failure, defined as the composite of cardiac death, myocardial infarction, or target vessel revascularization [48]. However, significant interactions for diabetic status and higher rates of stent thrombosis in the monotherapy arm raised concerns over the robustness of the non-inferiority claim, raising questions regarding a type I statistical error [48]. Further evidence from the ISAR-SAFE and SECURITY trials partially mitigated these concerns. Indeed, both trials reported non-inferiority of 6-month DAPT followed by aspirin monotherapy compared to standard DAPT for net adverse clinical events (NACE) at 12 months, defined as the composite of death, myocardial infarction, stent thrombosis, stroke, or major bleeding, among 4005 and 1399 CAD patients revascularized through PCI, respectively [51,53]. In addition, 6-month DAPT also resulted in a 59% reduction in any bleeding compared to prolonged DAPT in the ISAR-SAFE trial [51]. However, both trials were terminated prematurely due to slow recruitment and lower than expected event rates, suggesting possible bias and reduced power in the overall non-inferiority evaluation [67]. Finally, the ITALIC trial randomized 2031 patients to receive 6- or 24-month DAPT followed by aspirin monotherapy, with shorter DAPT duration being deemed non-inferior to prolonged DAPT for NACE, without significant differences for either ischemic or bleeding endpoints [52].

In light of these findings, further research on aspirin monotherapy focused on even shorter DAPT duration (i.e., <6 months). In the RESET and OPTIMIZE trials, 2117 and 3211 CAD patients undergoing PCI were randomized to either receive 3-month DAPT followed by aspirin monotherapy or 12-month DAPT, with both trials concluding that 3-month DAPT was non-inferior to longer DAPT duration for NACE at 12 months [49,50]. Finally, the One-Month DAPT trial further reduced the overall DAPT duration; 3020 patients with CAD undergoing PCI were randomized to either 1-month DAPT followed by aspirin monotherapy or standard DAPT duration, finding similar rates of ischemic and bleeding events between the two DAPT durations, establishing the non-inferiority of 1-month DAPT for NACE compared to standard DAPT [54]. However, the use of aspirin monotherapy after less than 6-month DAPT was associated with numerically higher rates of ischemic events, particularly in the setting of ACS, limiting its application to patients deemed at high risk of bleeding.

Aiming to derive final conclusions on the optimal DAPT duration, a systematic review and meta-analysis of 10 trials and 31,666 patients compared less than 6-month DAPT with 12-month or longer DAPT, finding that, although 12-month or longer DAPT duration was associated with reduced rates of myocardial infarction, DAPT of 6 months or less resulted in a 28% reduction in all-cause mortality, more likely due to the reduced incidence of fatal bleeding, as suggested by the concomitant 42% reduction in major bleeding reported by the authors [68]. On this basis, current guidelines recommend administration of 6-month DAPT in most patients presenting with stable CAD undergoing PCI, while longer DAPT durations are recommended in specific high-risk cases, such as patients with ACS or patients with complex procedures [2,3]. In addition, European guidelines give a class I recommendation for interrupting DAPT after 1 to 3 months in patients deemed at high bleeding risk, while giving a class IIb recommendation in patients without high ischemic nor bleeding risk [2]. Similarly, American guidelines recommend administering DAPT for 6 months after PCI in stable patients, while giving a class IIb recommendation for 1-month DAPT followed by aspirin monotherapy in ACS patients deemed at high bleeding risk [3,69].

### 4.2. Evidence and Recommendation on Short DAPT Followed by P2Y_12_ Receptor Inhibitor Monotherapy

The numerically higher rates of ischemic events reported with aspirin monotherapy raised some concerns for the overall safety of aspirin monotherapy, suggesting that enhanced antiplatelet potency should be obtained in order to achieve adequate ischemic protection after short DAPT while preventing unnecessary bleeding. Pharmacodynamic data suggest that P2Y_12_ inhibitor monotherapy may overcome the limitation of aspirin monotherapy, especially when more potent P2Y_12_ receptor inhibitors are administered [28]. Indeed, aspirin withdrawal appears to be associated with increased platelet reactivity driven by COX-mediated pathways, while maintaining P2Y_12_- and thrombin receptor-activated pathways inhibited [28], but this does not translate into increased blood thrombogenicity [70]. Accordingly, these pharmacodynamic findings have fueled interest in exploring strategies of P2Y_12_ inhibitor monotherapy following PCI.

Initial data evaluating the relative benefits of clopidogrel monotherapy after a short course of DAPT compared to prolonged DAPT came from the PRODIGY and STOPDAPT-2 trials. In the PRODIGY trial, 2013 patients undergoing PCI who completed 1-month DAPT were randomly assigned to either additional 6-month DAPT followed by clopidogrel monotherapy or 24 months of DAPT; it was found that clopidogrel monotherapy was associated with overall similar rates of major adverse cardiovascular events (MACE) while reducing major bleedings by 44%, independently of the definition applied [55]. Similarly, 1-month DAPT followed by clopidogrel monotherapy was non-inferior to standard DAPT for NACE among 5997 CAD patients undergoing PCI in the STOPDAPT-2 trial [59]. In addition, clopidogrel monotherapy reduced bleeding events by 62% compared to longer-term DAPT [59]. However, a significant increase in myocardial infarction was detected in the ACS population [71], questioning whether the higher degree of platelet inhibition achieved with a more potent P2Y_12_ receptor inhibitor may achieve better long-term prognosis in the context of monotherapy. The ongoing GENOSS DAPT (NCT05770674) trial will provide further insights into the usefulness of clopidogrel monotherapy after a short course of DAPT.

Among the available potent P2Y_12_ receptor inhibitors, ticagrelor monotherapy has been extensively evaluated after a short course of DAPT, while limited evidence exists on prasugrel monotherapy. Although initial data from the GLOBAL LEADERS trial showed no significant difference between 1-month DAPT followed by ticagrelor monotherapy or 12-month ticagrelor-based DAPT followed by aspirin monotherapy [56], supportive evidence came from a subsequent trial focusing on patients deemed at higher risk for bleeding or ischemic events [58]. In particular, in the TWILIGHT trial, 3-month DATP followed by ticagrelor monotherapy resulted in a 44% reduction for the primary endpoint of any bleeding, including a 51% reduction in major bleeding compared to standard ticagrelor-based DAPT among 7119 patients revascularized through PCI at higher risk for thrombotic or bleeding events (i.e., having at least one among the clinical criteria of age > 65 years, female sex, elevated troponin, previous evidence of systemic atherosclerosis, diabetes mellitus, and chronic kidney disease (CKD) and one among the angiographic criteria of multivessel disease, stent length >30 mm, thrombotic lesion, left main or anterior descending artery involvement, or calcified lesion requiring atherectomy) at 1 year [58]. Of note, this benefit came without significant trade-offs in ischemic events [58]. Similarly, more consistent results in support of ticagrelor monotherapy are available for the subset of ACS patients. In the TICO trial, 3056 patients with ACS were randomized to 3-month DAPT followed by ticagrelor monotherapy or 12-month DAPT, with the shorter DAPT regimen reducing NACE by 34%, being primarily driven by a 44% reduction in major bleeding [57]. Similarly, the more recent T-PASS and ULTIMATE DAPT trials randomized patients to receive up to 1-month DAPT followed by ticagrelor monotherapy or 12-month DAPT, and in both cases, shorter DAPT duration was associated with a significant reduction in NACE (46% and 32%, respectively), being driven by a reduction in major bleeding (reported as 65% and 61% reduction, respectively) without a significant increase in ischemic events [60,61]. Interestingly, results of the REC-CAGEFREEII trial suggest that the advantages of ticagrelor monotherapy in ACS patients are maximal if monotherapy is continued for at least 12 months. Indeed, in the REC-CAGEFREEII trial, 5-month ticagrelor monotherapy followed by subsequent aspirin monotherapy was non-inferior for NACE among 1948 ACS patients treated with PCI with drug-coated balloons, but a 1.4% significantly higher risk of ischemic events among patients in the monotherapy group was reported [72]. Among the ongoing trials exploring shorter duration of DAPT (Table 2), the BULK-STEMI (NCT04570345) trial will provide further evidence on ticagrelor monotherapy after a short period of DAPT compared to standard DAPT.

There is very limited data on prasugrel monotherapy, and current evidence derives from three trials giving mixed results. Although evidence from the ASET pilot study showed that aspirin discontinuation the day after PCI followed by prasugrel monotherapy was safe in a cohort of 426 patients with chronic coronary syndrome and non-complex disease undergoing PCI [73], the large-scale STOPDAPT-3 raised concerns over the general safety of such a strategy [74]. Specifically, prasugrel 3.75 mg monotherapy immediately after the index PCI was non-inferior to a standard DAPT regimen for the endpoint of major bleeding, while raising safety concerns due to a significant increase in repeat revascularization at 1-month follow-up among 5966 patients with ACS or high bleeding risk undergoing PCI [74]. However, these differences were no longer significant at 1 year, when P2Y_12_ inhibitor and aspirin monotherapies had comparable rates of ischemic and bleeding events, suggesting that the higher risk of revascularization detected during the first month was more likely due to the higher thrombotic risk associated with the initial periprocedural period [75]. Conversely, supportive data for prasugrel monotherapy were shown in the 4D-ACS trial, where 656 ACS patients were randomized to either 12-month DAPT or 1-month DAPT followed by prasugrel monotherapy [76]. Of note, after the initial month of standard DAPT during which prasugrel 10 mg was given, all patients received a 5 mg dose of prasugrel. At 12 months, prasugrel monotherapy resulted in a 49% reduction in NACE, being primarily driven by a 87% reduction in major bleeding, mostly of gastrointestinal origin [76]. On this background, it is plausible to assume that, if prasugrel monotherapy is planned, aspirin should still be given at least during the first month, and a 10 mg dosage should be used to avoid increased risk of ischemic events. Further evidence on prasugrel monotherapy will be provided by the ongoing COMPARE STEMI ONE (NCT05491200) [77], SMART-CHOICE 4 (NCT05066789), and SORT OUT DAPT (NCT06718179) trials, which are currently exploring the efficacy and safety of prasugrel monotherapy after 1 month of DAPT compared to standard DAPT.

Pooled data from a study-level meta-analysis encompassing 27,284 patients from seven randomized controlled trials showed that P2Y_12_ inhibitor monotherapy was associated with a 25% reduction in NACE, being driven by a 46% and 53% reduction in any bleeding and major bleeding, respectively [78]. However, a significant interaction for the type of P2Y_12_ inhibitor administered was detected, as ticagrelor monotherapy was the drug actually driving the results for NACE, while clopidogrel was associated with higher rates of ischemic endpoints and an 87% increase in myocardial infarction [78]. In addition, clopidogrel monotherapy was also associated with a higher risk of death compared to standard DAPT in the per-protocol analysis of a patient-level data meta-analysis, while ticagrelor monotherapy seemed to reduce mortality by 28% [79]. Of note, none of the available meta-analyses actually included trials of prasugrel, so these results should be limited to ticagrelor and clopidogrel.

While European guidelines on CCS patients do not give a specific recommendation for preferring P2Y_12_ inhibitor or aspirin monotherapy after a short period of DAPT in the early post-PCI phase [2], American guidelines give a class IIa recommendation for P2Y_12_ inhibitor monotherapy after 1–3 months of DAPT [3]. Regarding ACS patients, current European guidelines give a class IIa recommendation on DAPT discontinuation after 3 to 6 months, stating that P2Y_12_ inhibitors should be preferred over aspirin for subsequent monotherapy, while giving a class IIb recommendation for discontinuing aspirin after 1-month DAPT [80]. Instead, American guidelines on ACS give a class I recommendation for transitioning to ticagrelor monotherapy after 1 month of DAPT, while giving a class IIb for transitioning to other monotherapies after the same period of time in patients at high bleeding risk [69].

However, it is important to mention that the different classes of recommendation are based on indirect comparisons and inferred from the overall evidence supporting one antiplatelet agent over another, as better quality evidence is available for studies of P2Y_12_ inhibitor monotherapy rather than aspirin monotherapy (Table 3), while there is currently no trial directly comparing antiplatelet monotherapies after a short course of DAPT. The ongoing MODE-C trial (NCT05320926) is directly comparing two antiplatelet monotherapy strategies after a short course of DAPT, randomizing patients to receive clopidogrel or aspirin monotherapy after 1–3 months of DAPT. Evidence from these trials will allow current guidelines to implement specific recommendations on the specific drugs to be administered in the context of short DAPT across the spectrum of CAD undergoing PCI.

Currently, there are no recommendations from guidelines or regulatory agencies on using an “aspirin-free” strategy immediately after PCI. However, ongoing studies are exploring such aspirin-free strategies compared to standard DAPT, namely, the PREMIUM (NCT05709626) and PROMOTE (NCT06916520) trials, expanding on the current findings from the STOPDAPT-3 trial. Conversely, the TICALONE (NCT06509893) and TIMO (NCT05149560) trials will evaluate the efficacy of ticagrelor monotherapy, with the TIMO trial focusing on patients undergoing intravascular optical coherence tomography-guided PCI. Finally, the NEOMINDSET (NCT04360720) trial will evaluate the general usefulness of potent P2Y_12_ receptor inhibitor monotherapy, without specifying which antiplatelet drug should be preferred between prasugrel or ticagrelor [81].

## 5. Choice of Antiplatelet Therapy for Long-Term Secondary Prevention

**Figure 3:**
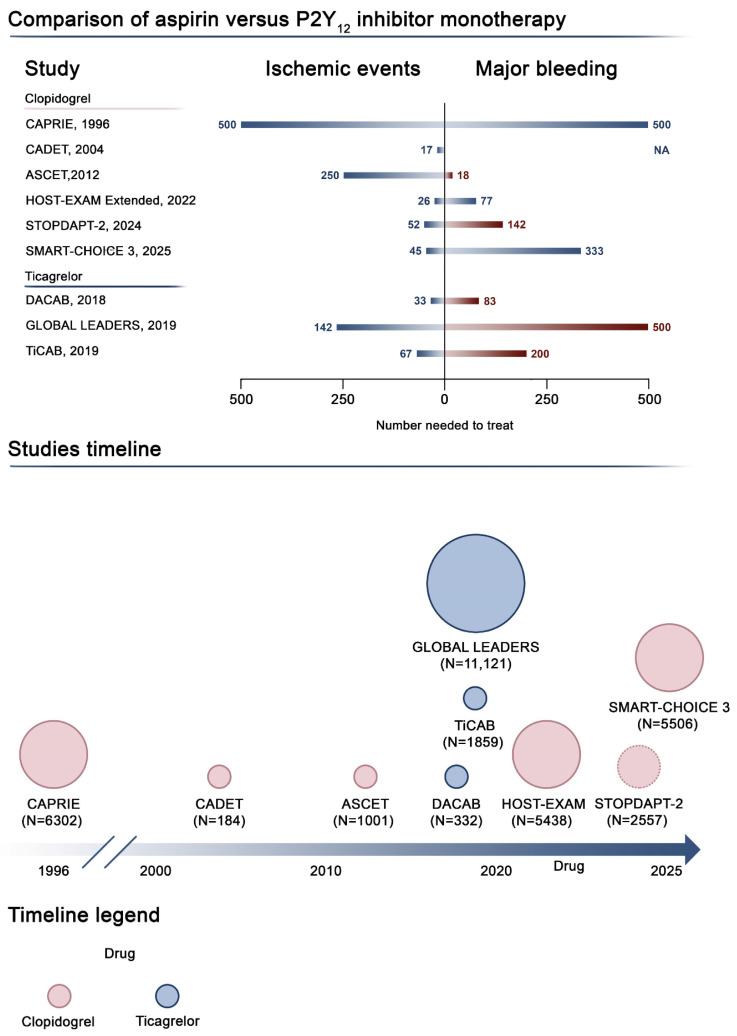
P2Y_12_ inhibitor monotherapy compared with aspirin monotherapy. This figure illustrates the current evidence on antiplatelet monotherapy compared with aspirin monotherapy. The top panel shows the number needed to treat for benefit (blue bars) or harm (red bars) for ischemic endpoints and major bleeding across studies. The timeline at the bottom illustrates current available studies exploring P2Y_12_ inhibitor monotherapy compared with aspirin monotherapy. The dimensions of the circles are proportional to the number of patients enrolled, while colors highlight the specific P2Y_12_ inhibitor administered as monotherapy. Abbreviations: CI, confidence interval; DAPT, dual antiplatelet therapy; HR, hazard ratio; NA, not available; P2Y12-i, P2Y12 inhibitor [82,83,84,85,86,87,88,89,90].

In addition to defining the optimal DAPT duration and which monotherapy regimen to use in patients undergoing PCI, particularly within the first year, another key issue is the choice of antiplatelet drug to administer for long-term prevention of recurrent ischemic events. While aspirin has been for decades the treatment of choice for long-term secondary prevention in CAD, nine trials have investigated the relative benefits of clopidogrel and ticagrelor over aspirin monotherapy, giving mixed results (Figure 3, Table 4).

### 5.1. Clopidogrel

Clopidogrel was the first P2Y_12_ inhibitor tested against aspirin in the context of monotherapy in patients with CAD, and the interest in clopidogrel monotherapy was further increased after the recognition that its administration is associated with a better risk–benefit profile compared to aspirin in patients at high risk for bleeding, such as those receiving oral anticoagulants [91]. Overall, six trials compared the clopidogrel and aspirin monotherapies in patients with CAD.

The first trial exploring this possible comparison was the CAPRIE trial, which randomized 19,185 patients at high risk for ischemic events (i.e., patients with history of myocardial infarction, stroke, or peripheral artery disease) to either receive clopidogrel 75 mg od or aspirin 325 mg od, finding a significant 8.7% relative reduction in the composite of vascular death, ischemic stroke, or myocardial infarction for clopidogrel compared to aspirin after a mean follow-up of 1.9 years [82]. However, a significant interaction for the baseline enrollment characteristic was found, as the effects were most beneficial in patients with peripheral arterial disease and stroke, while patients with myocardial infarction had overall neutral results.

These results highlighted that further pharmacodynamic data and patient characterization were necessary to identify those patients who may benefit more from clopidogrel rather than aspirin, leading to the development of the first small-sample trial aiming to investigate possible differences between the two drugs. The CADET trial showed that clopidogrel had similar in vitro effects in indices of thrombosis in 184 patients with myocardial infarction, as well as similar ischemic outcomes, although it was not powered to detect differences in hard clinical endpoints [83]. Similarly, the ASCET trial randomized 1001 patients with stable CAD to receive clopidogrel 75 mg od or aspirin 160 mg od, finding that the two drugs had similar rates of MACE, but clopidogrel was associated with a lower rate of bleeding at 2 years, being primarily driven by minor bleeding [84].

Although these preliminary data suggested a better risk–benefit profile for clopidogrel compared to aspirin monotherapy, this needed to be supported by large-scale randomized trials. To this extent, two large-scale trials, namely, the HOST-EXAM and SMART-CHOICE 3 trials, were conducted [85,86]. In the HOST-EXAM trial, clopidogrel monotherapy resulted in a 27% relative reduction in 2-year NACE compared to aspirin monotherapy among 5530 patients with CAD revascularized through PCI and who completed a standard DAPT regimen [85], being driven by a 32% reduction in MACE and a 36% reduction in major bleeding. Consistent results were detected when the follow-up was extended up to 5 years, with clopidogrel monotherapy resulting in a 27% reduction in NACE, a 34% reduction in MACE, and a 35% reduction in major bleeding [92]. Similarly, the SMART-CHOICE 3 trial randomized 5542 patients with CAD revascularized through PCI who completed a standard DAPT regimen to either receive aspirin or clopidogrel monotherapy [86], finding a 22% reduction in NACE and a 29% reduction in MACE with clopidogrel, primarily driven by reduction in myocardial infarction, while bleeding rates were generally similar between the two groups, although a significant reduction in bleeding of gastrointestinal origin was noted.

Finally, further evidence on the efficacy and safety of clopidogrel compared to aspirin monotherapy derives from a sub-analysis from the STOPDAPT-2 trial, which randomized 3045 patients undergoing PCI to either receive 1-month DAPT followed by clopidogrel monotherapy or 12-month DAPT followed by aspirin monotherapy [87]. In a landmark analysis between 12 and 60 months, where all patients received clopidogrel and aspirin monotherapy, clopidogrel was shown to be non-inferior to aspirin for both NACE and MACE. However, although clopidogrel was associated with a 39% reduction in myocardial infarction, it led to a 43% increase in any bleeding compared to aspirin.

In aggregate, these results suggest that clopidogrel may achieve an overall better efficacy/benefit profile compared to aspirin. Indeed, based on the available evidence, current European guidelines give a class I recommendation to administer clopidogrel in patients with previous myocardial infarction or remote PCI as a safe and effective alternative to aspirin monotherapy [2]. Conversely, American guidelines state that clopidogrel monotherapy may be used as an alternative to low-dose aspirin in individuals who cannot tolerate aspirin therapy but without giving a specific class of recommendation [3,93].

### 5.2. Ticagrelor

Given the higher antiplatelet potency of ticagrelor compared to clopidogrel, and the mounting evidence that ticagrelor monotherapy may achieve a better risk–benefit profile compared to clopidogrel monotherapy following short DAPT duration [78], ticagrelor monotherapy has been hypothesized as a viable option to further improve long-term outcomes in CAD patients.

Two studies compared the two monotherapy strategies in patients undergoing CABG, namely, the DACAB and TiCAB trials [88,89]. In the DACAB trial, 500 patients were randomized in a 1:1:1 ratio to either receive ticagrelor-based DAPT, ticagrelor monotherapy, or aspirin monotherapy within 24 h after CABG, showing that the three antiplatelet regimens had overall similar results for ischemic and bleeding endpoints, although ticagrelor-based DAPT was associated with the highest rates of graft patency at 1 year [88]. Similarly, 1859 patients undergoing CABG were enrolled in the TiCAB trial, being randomized to either receive ticagrelor or aspirin monotherapy, finding overall neutral results for the primary endpoint of MACE at 1 year, as well as similar rates of bleeding events between the two cohorts [89]. Of note, the study was halted prematurely due to higher-than-expected event rates in the ticagrelor group and a lack of funding, leading to an overall underpowered analysis.

Regarding patients revascularized through PCI, there is limited evidence supporting the use of ticagrelor monotherapy, mostly deriving from sub-analyses of randomized controlled trials. Specifically, the only available data on ticagrelor monotherapy derive from a landmark study of the GLOBAL LEADERS trial. Indeed, landmark analysis between 12 and 24 months, where all patients received either aspirin or ticagrelor monotherapy, showed that ticagrelor monotherapy significantly reduced MACE by 26%, being primarily driven by reduced rates of myocardial infarction, at the expense of a 47% increased risk of any bleeding, including major bleeding [90]. Given the lack of sufficient evidence to support administration of ticagrelor monotherapy in the context of long-term prevention in patients with CAD, European guidelines give a class IIb recommendation for ticagrelor monotherapy in CCS and stabilized ACS patients as an alternative to other antiplatelet strategies, while American guidelines do not give specific recommendations on its use [2,3].

Aiming to overcome the limitations of single randomized controlled trials in order to derive definitive evidence on the use of P2Y_12_ receptor inhibitor monotherapy over aspirin monotherapy, these trials were pooled together in an individual patient data meta-analysis, sowing that P2Y_12_ receptor inhibitor monotherapy resulted in a 11% reduction in NACE and a 12% reduction in MACE [94]. Interestingly, bleeding appears to be reduced by P2Y_12_ receptor inhibitor monotherapy, mainly driven by a 25% reduction in gastrointestinal bleeding and a 57% reduction in hemorrhagic stroke, without interactions with respect to the type of P2Y_12_ inhibitor administered (i.e., clopidogrel or ticagrelor). Although a major limitation of this analysis was the absence of specific data for the type of revascularization, a subsequent patient-level data meta-analysis focusing on patients undergoing PCI retrieved consistent results [95]. Of note, the data supporting this evidence are overall of moderate–high quality, with better quality evidence available for clopidogrel rather than ticagrelor monotherapy (Table 5), so these recommendations should be referred mostly to clopidogrel rather than ticagrelor monotherapy.

### 5.3. Limitations of P2Y_12_ Inhibitor Monotherapy

Although mounting evidence supports the use of P2Y_12_ inhibitor monotherapy as a secondary prevention strategy instead of aspirin, several considerations need to be made.

The pharmacodynamic effects of clopidogrel are characterized by substantial interindividual variability [32], which may have influenced outcomes of randomized controlled trials comparing clopidogrel to aspirin. Indeed, aspirin resistance is less common than clopidogrel resistance, with the latter occurring in as much as 30% of patients [96]. Therefore, the predictable antiplatelet effects of aspirin may make aspirin a wise choice for long-term monotherapy when genetic or platelet function tests are not available for determining if clopidogrel is achieving its therapeutic effects.

Another option to overcome the interindividual variability of clopidogrel could be the administration of more potent P2Y_12_ inhibitors, such as prasugrel or ticagrelor. However, while their administration as monotherapies in the ACS setting after a short period of DAPT has shown remarkable improvement in long-term outcomes [76,78,95], the increased rates of bleeding in the long-term may overcome the benefits derived from ischemic protection [17]. The administration of lower dosages of these drugs as monotherapies has shown promising results in pharmacodynamic studies [28], but their efficacy in terms of hard clinical endpoints has not yet been evaluated. Given these uncertainties, current international consensus on the use of platelet function and genetic testing in patients undergoing PCI recommends using either platelet function or genetic testing in those patients selected to receive P2Y_12_ inhibitor monotherapy deemed at high ischemic risk, such as those with a history of recurrent ischemic events [38]. Based on the results of these tests, clopidogrel responders should receive clopidogrel monotherapy, while non-responders should receive either ticagrelor or prasugrel monotherapy.

Additionally, drug-specific adverse events related to P2Y_12_ inhibitors, such as the high rates of dyspnea seen with ticagrelor and higher rates of nuisance bleeding seen with more potent P2Y_12_ inhibitors [97,98], may lead to abrupt drug discontinuation and unfavorable outcomes [65]. On the other hand, clopidogrel and aspirin are associated with fewer adverse events, potentially having higher adherence among patients and being more manageable in the long-term [93].

Along with the aforementioned limitations, the perioperative management of antiplatelet agents in patients undergoing major surgery may be challenging. Indeed, while supportive evidence for periprocedural administration of aspirin monotherapy in patients with previous PCI can be derived from a subgroup analysis of the POISE-2 trial, where aspirin was shown to reduce MACE by 50% compared to placebo among 470 patients undergoing noncardiac surgery [99], there is currently no data on the periprocedural administration of P2Y_12_ inhibitors, and most surgeons remain hesitant to operate on patients receiving P2Y_12_ inhibitors, frequently requiring patients to switch to aspirin for their procedure [100].

**Figure 4:**
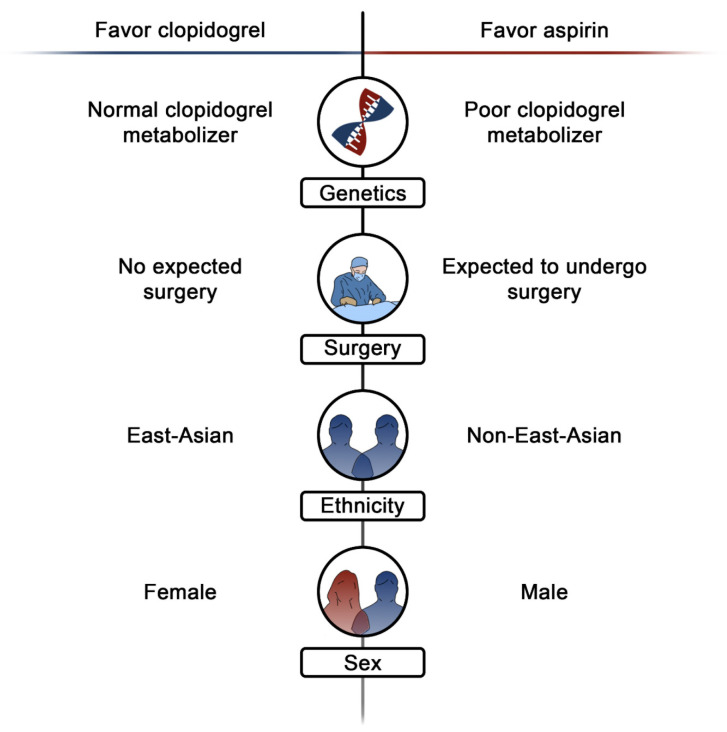
**Patient characteristics favoring aspirin or clopidogrel monotherapy for long-term secondary prevention.** This figure illustrates practical implications that should be considered when choosing between clopidogrel (left) or aspirin (right) monotherapy, according to the current literature supporting the use of these drugs.

**Figure 5:**
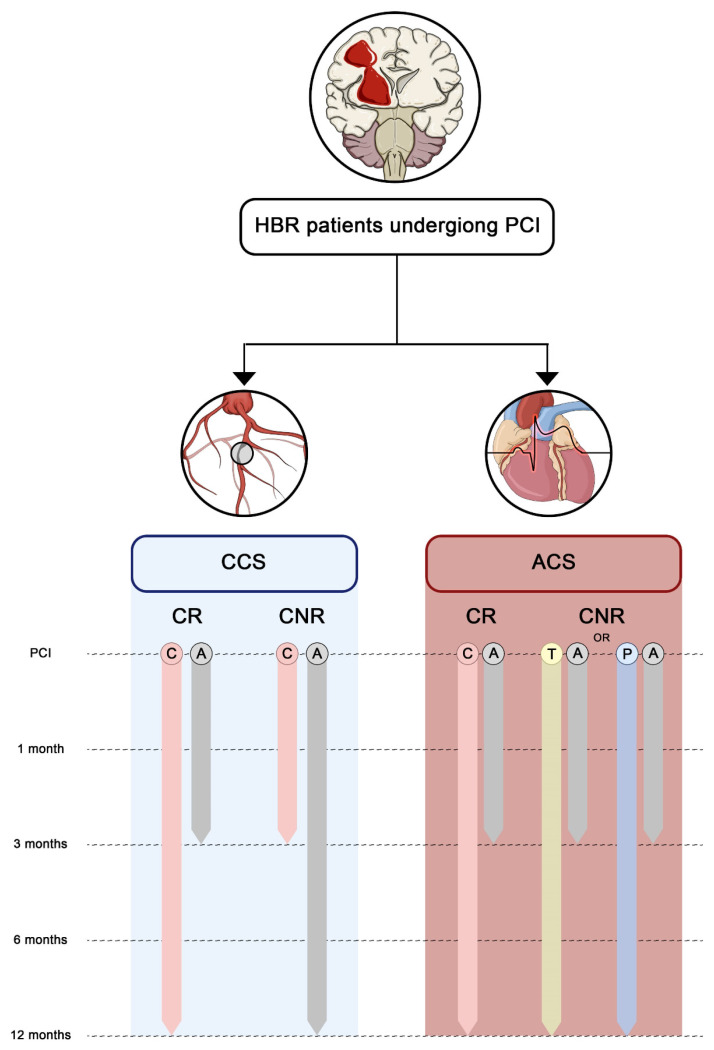
**Framework for antiplatelet monotherapy selection in patients at high bleeding risk and undergoing PCI, depending on presentation and responsiveness to clopidogrel.** This figure illustrates a framework for selecting the most appropriate antiplatelet monotherapy regimen in HBR patients undergoing PCI depending on clinical presentation and clopidogrel responsiveness. After the initial high-thrombotic-risk period (i.e., the first 3 months), CCS patients at HBR may benefit from clopidogrel monotherapy. However, this benefit is maximal only in patients deemed to be clopidogrel responders by either genetic or platelet function testing. In CNR patients, however, clopidogrel may not be suitable for the prevention of ischemic events, and aspirin monotherapy should be adopted instead. In ACS patients, instead, ticagrelor 90 mg or prasugrel 10 mg monotherapies should be preferred, with the exception of CR patients, where the antithrombotic potency achieved with clopidogrel may provide an optimal balance between ischemic and bleeding risk. Abbreviations: A, aspirin; ACS, acute coronary syndrome; C, clopidogrel; CCS, chronic coronary syndrome; CNR, clopidogrel non-responder; CR, clopidogrel responder; HBR, high bleeding risk; P, prasugrel; PCI, percutaneous coronary intervention; T, ticagrelor.

Finally, since most of the supportive data on P2Y_12_ inhibitor monotherapy derives from trials focusing on East Asian patients [101], the generalizability to other ethnicities may be limited, as East Asian patients tend to have a higher tendency toward bleeding compared to other populations [102]. Similarly, there is substantial imbalance in the proportion of male and female patients enrolled in trials of antiplatelet monotherapies, and significant sex-related interactions have been detected previously, with female patients obtaining a higher benefit from P2Y_12_ inhibitor monotherapy compared to male patients, likely due to the higher baseline risk for bleeding and higher baseline P2Y_12_ receptor activity [103]. In addition, there is a significant lack of data from real-world registries, as DAPT modulation strategies are significantly underused compared to actual recommendations [104,105]. Figure 4 summarizes patient characteristics that may favor aspirin or clopidogrel monotherapy for long-term secondary prevention of ischemic events, while Figure 5 provides a framework for antiplatelet monotherapy selection to adopt in the first 12 months in patients at high bleeding risk and undergoing PCI.

## 6. Special Populations

On top of the aforementioned characteristics that may guide decisions on the selection of antiplatelet monotherapy, several subgroup populations have been shown to have a higher baseline risk of thrombosis and bleeding compared to the standard CAD population undergoing PCI, including patients with CKD and patients with diabetes mellitus.

### 6.1. Patients with CKD

CKD patients represent a high-risk population, with rates of cardiovascular events being inversely correlated with renal function [106]. However, these patients are not only at high risk for ischemic events, but they are also at a high risk of bleeding, therefore representing a specific population where defining the optimal antiplatelet therapy is of the utmost importance to prevent long-term adverse events [107,108].

Regarding short DAPT followed by antiplatelet monotherapy, most of the available data on CKD patients derive from trials of P2Y_12_ inhibitor monotherapy, rather than aspirin monotherapy. Indeed, the only data available for aspirin monotherapy are represented by subgroup analyses from the one-month DAPT trial, which showed consistent results with the main analysis among patients with CKD [54]. Conversely, almost all trials on P2Y_12_ inhibitor monotherapy reported data on CKD patients. When evaluating data on clopidogrel monotherapy, while the PRODIGY study did not report a significant interaction for CKD status on the overall treatment effect [109], data from the STOPDAPT-2 trial actually reported a higher benefit from clopidogrel monotherapy compared to standard DAPT among patients with severe CKD, as this population achieved a 48% reduction in NACE, while patients without CKD did not achieve substantial benefit [71]. Similarly, data from trials focusing on ticagrelor monotherapy reported significant benefit for ticagrelor monotherapy among CKD patients, without significant interaction on treatment effect estimates [60,61,110,111]. Summing the clinical with the pharmacodynamic evidence, suggesting that CKD status reduces the production of clopidogrel active metabolites and, therefore, increases on-treatment platelet reactivity [112], it is possible to infer that ticagrelor monotherapy should be the optimal antiplatelet regimen in this population when a monotherapy strategy is planned.

Regarding monotherapy strategies for long-term secondary prevention beyond the first year after the procedure, data on the subgroup of patients with CKD are only available for clopidogrel monotherapy, while previous trials on ticagrelor monotherapy did not report on this specific population. Although data from both the HOST-EXAM and SMART-CHOICE 3 trials reported the absence of an interaction between clopidogrel monotherapy and CKD status, both trials reported that the benefits of clopidogrel over aspirin were no longer significant among CKD patients. Although this can be due to the reduced statistical power of the single subgroups, the hypothesis of lower efficacy of clopidogrel in this population cannot be ruled out [86,113]. However, no significant adverse events were reported either, suggesting that still other factors should be taken into consideration when choosing the antiplatelet agent to be administered in this specific patient population.

### 6.2. Patients with Diabetes Mellitus

Diabetes mellitus has long been known for increasing the risk of recurrent ischemic events in patients with CAD. Indeed, diabetic patients have a prothrombotic milieu represented by enhanced platelet reactivity, higher inflammation burden, and endothelial disfunction, leading to increased risk of ischemic events [114], and tailoring the selection of the antiplatelet regimen is required to accurately balance the increased ischemic risk of this population with the increased bleeding risk associated with the other comorbidities that are usually associated with this condition [115].

This increased ischemic risk resulted in significant interaction for the antiplatelet regimen administered in the original report from the EXCELLENT trial, where short DAPT followed by aspirin monotherapy resulted in a three-fold higher risk of ischemic events compared to 12-month DAPT in the subgroup of patients with diabetes, in contrast with the results of the main analysis [48]. Conversely, no significant interaction for diabetes status was reported among other trials of short DAPT followed by aspirin monotherapy, which also used shorter DAPT durations [49,50,54]. These different results may be the reflection of progressive improvement in PCI techniques and scaffold [47], having lower thrombogenicity, although pitfalls in trial designs driving the conclusions cannot be ruled out. Conversely, data on P2Y_12_ inhibitor monotherapy showed more consistent results, with all trials reporting no significant interaction between the benefit of P2Y_12_ inhibitor monotherapy and diabetes mellitus [60,61,116,117]. However, in patients receiving less than 1-month DAPT, the benefits of P2Y_12_ inhibitor monotherapy appear to be non-significant [60], suggesting that a longer DAPT duration (i.e., 3 months) before transitioning to antiplatelet monotherapy may be useful, given the higher baseline ischemic risk of this population.

Conversely, when evaluating long-term antiplatelet monotherapies beyond 1 year from the procedure, all studies reported consistent results between the effects of P2Y_12_ inhibition compared to aspirin monotherapy among diabetic patients, without significant interaction, suggesting that diabetic status should not be a major driver of decision when considering the long-term antiplatelet regimen [86,88,89,90,118].

## 7. Conclusions

Administration of antiplatelet drugs significantly improves outcomes in patients with CAD, and DAPT is the current standard regimen in patients undergoing PCI. However, continuous procedural, technical, and pharmacological improvements, along with the recognition that bleeding events have significant drawbacks for overall patient prognosis, have significantly reduced the need for long-term DAPT in favor of antiplatelet monotherapy. In the context of post-procedural management, there is mounting evidence supporting the use of P2Y_12_ inhibitor monotherapy rather than aspirin monotherapy after a short course of DAPT, as this strategy has been shown to reduce bleeding without any trade-off in ischemic events. P2Y_12_ inhibitor monotherapy is gaining attention compared to aspirin monotherapy for long-term secondary prevention of ischemic events. However, data are still limited, and aspirin still remains the cornerstone of long-term treatment in patients with CAD who have previously undergone PCI.

## Figures and Tables

**Table 1:** Trials investigating shortened DAPT durations in CAD patients undergoing PCI.

Trial, Year	Population	Sample Size	Investigation	Control	Ischemic Endpoint	Results for the Ischemic Endpoint	Major Bleeding Endpoint	Results for Bleeding Endpoint	Follow-Up (Months)
**Aspirin monotherapy after short DAPT**									
**EXCELLENT, 2012** [48]	CAD patients undergoing PCI	1443	6-month clopidogrel-based DAPT followed by aspirin monotherapy	12-month clopidogrel-based DAPT	MACE	4.8% vs. 4.3%	TIMI major bleeding	0.3% vs. 0.6%	12
**RESET, 2012** [49]	CAD patients undergoing PCI	2148	3-month clopidogrel-based DAPT followed by aspirin monotherapy	12-month clopidogrel-based DAPT	MACE	4.7% vs. 4.7%	TIMI major bleeding	0.2% vs. 0.6%	12
**OPTIMIZE, 2013** [50]	CAD patients undergoing PCI	3211	3-month clopidogrel-based DAPT followed by aspirin monotherapy	12-month clopidogrel-based DAPT	MACE	8.3% vs. 7.4%	Trial-defined	0.5% vs. 0.5%	12
**ISAR-SAFE, 2014** [51]	CAD patients revascularized through PCI who completed 6-month DAPT	4005	6-month clopidogrel-based DAPT	Aspirin monotherapy	MACE	1.5% vs. 1.3%	TIMI major bleeding	0.3% vs. 0.2%	9
**ITALIC, 2014** [52]	CAD patients undergoing PCI	1850	6-month clopidogrel-based DAPT followed by aspirin monotherapy	24-month clopidogrel-based DAPT	Myocardial infarction	0.7% vs. 0.4%	TIMI major bleeding	0.0% vs. 0.4%	12
**SECURITY, 2014** [53]	CAD patients undergoing PCI	1399	6-month clopidogrel-based DAPT followed by aspirin monotherapy	12-month clopidogrel-based DAPT	Myocardial infarction	3.1% vs. 2.6%	BARC type 3 to 5	0.7% vs. 1.1%	24
**One-Month DAPT, 2021** [54]	CAD patients undergoing PCI	3020	1-month clopidogrel-based DAPT followed by aspirin monotherapy	6- to 12-month clopidogrel-based DAPT	Myocardial infarction	1.1% vs. 1.5%	STEEPLE major bleeding	1.7% vs. 2.5%	12
**P2Y12 receptor inhibitor monotherapy**									
**PRODIGY, 2012** [55]	CAD patients undergoing PCI who completed 1-month DAPT	2013	6-month clopidogrel-based DAPT followed by clopidogrel monotherapy	24-month clopidogrel-based DAPT	MACE	10.0% vs. 10.1%	BARC type 3 or 5	1.9% vs. 3.4%	24
**GLOBAL LEADERS, 2018** [56]	CAD patients undergoing PCI	15,968	1-month ticagrelor-based DAPT followed by ticagrelor monotherapy	12-month ticagrelor-based DAPT followed by aspirin monotherapy	MACE	3.8% vs. 4.4%	BARC type 3 or 5	2.0% vs. 2.1%	24
**TICO, 2020** [57]	ACS patients undergoing PCI	3056	3-month ticagrelor-based DAPT followed by ticagrelor monotherapy	12-month ticagrelor-based DAPT	MACE	1.2% vs. 2.0%	TIMI major bleeding	1.7% vs. 3.0%	12
**TWILIGHT, 2020** [58]	CAD patients at high risk for ischemic or bleeding events undergoing PCI who completed 3-month ticagrelor-based DAPT	7119	Ticagrelor 90 mg monotherapy	Ticagrelor 90 mg-based DAPT	MACE	3.9% vs. 3.9%	BARC type 3 or 5	1.0% vs. 2.0%	12
**STOPDAPT-2, 2022** [59]	CAD patients undergoing PCI	5997	1-month clopidogrel-based DAPT followed by clopidogrel monotherapy	12-month clopidogrel-based DAPT	MACE	2.8% vs. 3.0%	TIMI major bleeding	0.3% vs. 0.8%	12
**T-PASS, 2023** [60]	ACS patients undergoing PCI	2850	Up to 1-month ticagrelor-based DAPT followed by ticagrelor monotherapy	12-month ticagrelor-based DAPT	MACE	1.8% vs. 2.2%	BARC type 3 to 5	1.2% vs. 3.4%	12
**ULTIMATE DAPT, 2024** [61]	ACS patients undergoing PCI	3400	1-month clopidogrel-based DAPT followed by ticagrelor monotherapy	12-month ticagrelor-based DAPT	MACE	3.6% vs. 3.7%	BARC type 3 or 5	0.7% vs. 1.7%	12

Outcomes are reported as investigation vs. control. Abbreviations: ACS, acute coronary syndrome; BARC, bleeding academic research consortium; CAD, coronary artery disease; DAPT, dual antiplatelet therapy; MACE, major adverse cardiovascular events; PCI, percutaneous coronary intervention; TIMI, thrombolysis in myocardial infarction.

**Table 2:** Ongoing trials focusing on antiplatelet monotherapies.

Trial, NCT	Sample (n)	Condition	Intervention	Control	Primary Endpoint
**Reduced DAPT duration followed by antiplatelet monotherapy**					
**AGIODAPT, NCT05952206**	2312	CAD patients undergoing PCI	1-month DAPT followed by P2Y_12_ receptor inhibitor monotherapy	12-month DAPT	BARC 2, 3, or 5
**BULK-STEMI, NCT04570345**	1002	STEMI patients undergoing PCI	3-month DAPT followed by ticagrelor monotherapy	12-month DAPT	NACE
**CAGEFREEII, NCT04971356**	1948	ACS patients undergoing PCI with drug-coated balloon	1-month DAPT followed by 5-month ticagrelor monotherapy and subsequent aspirin monotherapy	12-month DAPT	NACE
**COMPARE STEMI ONE, NCT05491200**	1608	STEMI patients undergoing PCI	1-month DAPT followed by prasugrel monotherapy	12-month DAPT	MACE
**DUAL-ACS 2, NCT03252249**	4576	Type 1 myocardial infarction	3-month DAPT followed by antiplatelet monotherapy	12-month DAPT	All-cause death
**GENOSS DAPT, NCT05770674**	2186	CCS patients undergoing PCI	1-month DAPT followed by clopidogrel monotherapy	12-month DAPT	NACE
**MODE-C, NCT05320926**	3744	CCS patients undergoing PCI	1- to 3-month DAPT followed by clopidogrel monotherapy	1- to 3-month DAPT followed by aspirin monotherapy	NACE
**SMART-CHOICE 4, NCT05066789**	4000	ACS undergoing PCI	1-month DAPT followed by prasugrel monotherapy	12-month DAPT	MACE
**SORT OUT DAPT, NCT06718179**	3150	ACS patients undergoing PCI	1-month DAPT followed by prasugrel monotherapy	12-month DAPT	BARC 2, 3, or 5
**SHORTDAPT IVUS, NCT06648720**	3566	CAD patients undergoing intravascular ultrasound-guided PCI	1-month DAPT followed by P2Y_12_ receptor inhibitor monotherapy	12-month DAPT	NACE
**TARGET-FIRST, NCT04753749**	2248	ACS patients undergoing PCI	1-month DAPT followed by antiplatelet monotherapy	12-month DAPT	NACE
**P2Y_12_** **inhibitor monotherapy compared with DAPT**					
**NEOMINDSET, NCT04360720**	3400	ACS patients undergoing PCI	12-month potent P2Y_12_-inhibitor monotherapy	12-month DAPT	MACE
**PREMIUM, NCT05709626**	2258	STEMI undergoing PCI	12-month prasugrel monotherapy	12-month DAPT	MACE
**PROMOTE, NCT06916520**	300	CAD patients undergoing PCI	12-month prasugrel monotherapy	12-month DAPT	NACE
**STOP-IMH, NCT05986968**	200	Type 1 myocardial infarction patients undergoing PCI	12-month ticagrelor monotherapy	12-month DAPT	MACE
**TICALONE, NCT06509893**	5400	CCS patients undergoing PCI	6-month ticagrelor monotherapy	6-month DAPT	MACE
**TIMO, NCT05149560**	200	ACS patients undergoing optical-coherence tomography-guided PCI	3-month ticagrelor monotherapy	3-month DAPT	MACE

Abbreviations: ACS, acute coronary syndrome; BARC, Bleeding Academic Research Consortium; CAD, coronary artery disease; CCS, chronic coronary syndrome; DAPT, dual antiplatelet therapy; MACE, major adverse cardiac events; NACE, net adverse clinical event; NCT, National Clinical Trial number; PCI, percutaneous coronary intervention; SAPT, single antiplatelet therapy; STEMI, ST-segment elevated myocardial infarction.

**Table 3:** Grading quality of evidence and strength of recommendations for studies of short DAPT followed by antiplatelet monotherapy.

Number of Studies	Risk of Bias	Inconsistency	Indirectness	Imprecision	Overall Quality
**All-cause death (aspirin monotherapy)**					
7	Serious	Not serious	Not serious	Not serious	⨁⨁
					Low
**All-cause death (P2Y_12_ inhibitor monotherapy)**					
7	Not serious	Not serious	Not serious	Not serious	⨁⨁⨁⨁
					High
**NACE (aspirin monotherapy)**					
5	Moderate	Moderate	Not serious	Not serious	⨁⨁⨁
					Moderate
**NACE (P2Y_12_ inhibitor monotherapy)**					
6	Not serious	Not serious	Not serious	Not serious	⨁⨁⨁⨁
					High
**MACE (aspirin monotherapy)**					
7	Serious	Moderate	Not serious	Not serious	⨁⨁
					Low
**MACE (P2Y_12_ inhibitor monotherapy)**					
7	Moderate	Moderate	Not serious	Not serious	⨁⨁⨁
					Moderate
**Myocardial infarction (aspirin monotherapy)**					
6	Moderate	Not serious	Not serious	Not serious	⨁⨁⨁
					Moderate
**Myocardial infarction (P2Y_12_ inhibitor monotherapy)**					
7	Not serious	Moderate	Not serious	Not serious	⨁⨁⨁
					Moderate
**Stroke (aspirin monotherapy)**					
7	Moderate	Not serious	Not serious	Not serious	⨁⨁⨁
					Moderate
**Stroke (P2Y_12_ inhibitor monotherapy)**					
7	Not serious	Not serious	Not serious	Not serious	⨁⨁⨁⨁
					High
**Stent thrombosis (aspirin monotherapy)**					
7	Moderate	Not serious	Not serious	Not serious	⨁⨁⨁
					Moderate
**Stent thrombosis (P2Y_12_ inhibitor monotherapy)**					
7	Not serious	Moderate	Not serious	Not serious	⨁⨁⨁
					Moderate
**Any bleeding (aspirin monotherapy)**					
7	Moderate	Not serious	Not serious	Not serious	⨁⨁⨁
					Moderate
**Any bleeding (P2Y_12_ inhibitor monotherapy)**					
7	Moderate	Not serious	Not serious	Not serious	⨁⨁⨁
					Moderate
**Major bleeding (aspirin monotherapy)**					
5	Moderate	Not serious	Not serious	Not serious	⨁⨁⨁
					Moderate
**Major bleeding (P2Y_12_ inhibitor monotherapy)**					
7	Not serious	Not serious	Not serious	Not serious	⨁⨁⨁⨁
					High

Abbreviations: DAPT, dual antiplatelet therapy; MACE, major adverse cardiovascular events; NACE, net adverse clinical events.

**Table 4:** Trials of P2Y_12_ receptor inhibitor monotherapy compared to aspirin monotherapy.

Trial, Year	Population	Sample Size	Investigation	Control	Ischemic Endpoint	Results for the Ischemic Endpoint	Major Bleeding Endpoint	Results for Bleeding Endpoint	Follow-Up (Months)
**Clopidogrel monotherapy**									
**CAPRIE, 1996** [82]	Previous myocardial infarction	6302	Clopidogrel 75 mg once daily	Aspirin 325 mg once daily	MACE	4.8% vs. 5.0%	Trial-defined	1.4% 1.6%	36
**CADET, 2004** [83]	Patients with myocardial infarction within 3 to 7 days	184	Clopidogrel 75 mg once daily	Aspirin 75 mg once daily	Myocardial infarction	1% vs. 7%	NR	NR	6
**ASCET, 2012** [84]	Angiographic evidence of stable CAD and high on-aspirin residual platelet reactivity	1001	Clopidogrel 75 mg once daily	Aspirin 160 mg once daily	MACE	10.4% vs. 10.8%	Trial-defined	15.8% vs. 10.2%	24
**HOST-EXAM Extended, 2022** [85]	CAD patients revascularized through PCI who completed 12-month DAPT	5438	Clopidogrel 75 mg once daily	100 mg once daily	MACE	8.1% vs. 11.9%	BARC type 3 or greater	2.6% vs. 3.9%	69.9
**STOPDAPT-2, 2024** [87]	CAD patient revascularized through PCI who completed 12-month antiplatelet regimen	2557	Clopidogrel 75 mg once daily	Aspirin 81 to 200 mg daily	MACE	6.8% vs. 8.7%	TIMI major bleeding	3.3% vs. 2.6%	48
**SMART-CHOICE 3, 2025** [86]	CAD patients revascularized through PCI who completed 6- or 12-month DAPT regimen depending on the presentation	5506	Clopidogrel 75 mg once daily	Aspirin 100 mg daily	MACE	4.4% vs. 6.6%	BARC type 3 or 5	1.6% vs. 1.3%	36
**Ticagrelor monotherapy**									
**DACAB, 2018** [88]	CAD patients revascularized through CABG	332	Ticagrelor 90 mg twice daily	Aspirin 100 mg daily	MACE	2.4% vs. 5.4%	Trial-defined	1.2% vs. 0.0%	12
**GLOBAL LEADERS, 2019** [90]	CAD patient revascularized through PCI who completed 12-month antiplatelet regimen	11,121	Ticagrelor 90 mg twice daily	Aspirin 75 to 100 mg daily	MACE	1.9% vs. 2.6%	BARC type 3 or 5	0.5% vs. 0.3%	24
**TiCAB, 2019** [89]	CAD patients revascularized through CABG	1859	Ticagrelor 90 mg twice daily	Aspirin 100 mg daily	MACE	9.7% vs. 8.2%	BARC type 3 to 5	3.7% vs. 3.2%	12

Outcomes are reported as investigation vs. control. Abbreviations: BARC, bleeding academic research consortium; CABG, coronary artery bypass graft; CAD, coronary artery disease; DAPT, dual antiplatelet therapy; MACE, major adverse cardiovascular events; PCI, percutaneous coronary intervention; TIMI, thrombolysis in myocardial infarction.

**Table 5:** Grading quality of evidence and strength of recommendations for long-term antiplatelet monotherapy beyond the first year post-index procedure.

Number of Studies	Risk of Bias	Inconsistency	Indirectness	Imprecision	Overall Quality
**All-cause death (clopidogrel monotherapy)**					
6	Not serious	Not serious	Not serious	Not serious	⨁⨁⨁⨁
					High
**All-cause death (ticagrelor monotherapy)**					
3	Not serious	Not serious	Not serious	Not serious	⨁⨁⨁⨁
					High
**NACE (clopidogrel monotherapy)**					
4	Moderate	Moderate	Not serious	Not serious	⨁⨁⨁
					Moderate
**NACE (ticagrelor inhibitor monotherapy)**					
2	Not serious	Moderate	Not serious	Not serious	⨁⨁⨁
					Moderate
**MACE (clopidogrel monotherapy)**					
6	Moderate	Not serious	Not serious	Not serious	⨁⨁⨁⨁
					High
**MACE (ticagrelor monotherapy)**					
3	Moderate	Moderate	Not serious	Not serious	⨁⨁⨁
					Moderate
**Myocardial infarction (clopidogrel monotherapy)**					
6	Moderate	Not serious	Not serious	Not serious	⨁⨁⨁
					Moderate
**Myocardial infarction (ticagrelor monotherapy)**					
3	Not serious	Moderate	Not serious	Not serious	⨁⨁⨁
					Moderate
**Stroke (clopidogrel monotherapy)**					
5	Not serious	Moderate	Not serious	Not serious	⨁⨁⨁
					Moderate
**Stroke (ticagrelor monotherapy)**					
2	Moderate	Not serious	Not serious	Not serious	⨁⨁⨁
					Moderate
**Stent thrombosis (clopidogrel monotherapy)**					
6	Not serious	Not serious	Not serious	Not serious	⨁⨁⨁
					Moderate
**Stent thrombosis (ticagrelor monotherapy)**					
1	Moderate	Moderate	Not serious	Not serious	⨁⨁
					Low
**Any bleeding (clopidogrel monotherapy)**					
6	Moderate	Not serious	Not serious	Not serious	⨁⨁⨁
					Moderate
**Any bleeding (ticagrelor monotherapy)**					
3	Moderate	Not serious	Not serious	Not serious	⨁⨁⨁
					Moderate
**Major bleeding (clopidogrel monotherapy)**					
4	Moderate	Not serious	Not serious	Not serious	⨁⨁⨁
					Moderate
**Major bleeding (ticagrelor monotherapy)**					
2	Moderate	Not serious	Not serious	Not serious	⨁⨁⨁
					Moderate

Abbreviations: DAPT, dual antiplatelet therapy; MACE, major adverse cardiovascular events; NACE, net adverse clinical events.

## Data Availability

All data are available upon reasonable request to the corresponding author.

## Abbreviations

ACS = Acute coronary syndrome; ADP = Adenosine diphosphate; CAD= Coronary artery disease; CCS = Chronic coronary syndrome; COX = Cyclooxygenase; CYP = Cytochrome P450; DAPT = Dual antiplatelet therapy; MACE = Major adverse cardiovascular events; NACE = Net adverse clinical events; NNT = Number needed to treat; PCI = Percutaneous coronary intervention.
